# hrHPV E5 oncoprotein: immune evasion and related immunotherapies

**DOI:** 10.1186/s13046-017-0541-1

**Published:** 2017-05-25

**Authors:** Antonio Carlos de Freitas, Talita Helena Araújo de Oliveira, Marconi Rego Barros, Aldo Venuti

**Affiliations:** 1 Department of Genetics, Laboratory of Molecular Studies and Experimental Therapy (LEMTE), Center of Biological Sciences, Federal University of Pernambuco, Av. Prof Moraes Rego, 1235, Cidade Universitária, Recife, CEP 50670-901 Brazil; 2 Department of Research, HPV-Unit, UOSD Tumor Immunology and Immunotherapy Unit, Advanced Diagnostic and Technological Innovation, Regina Elena National Cancer Institute, Via Elio Chianesi 53, 00144 Rome, Italy

**Keywords:** Human Papillomavirus, HPV-related cancer, E5 oncoprotein, Immune response modulation, HPV immune evasion, Immunotherapy

## Abstract

The immune response is a key factor in the fight against HPV infection and related cancers, and thus, HPV is able to promote immune evasion through the expression of oncogenes. In particular, the E5 oncogene is responsible for modulation of several immune mechanisms, including antigen presentation and inflammatory pathways. Moreover, E5 was suggested as a promising therapeutic target, since there is still no effective medical therapy for the treatment of HPV-related pre-neoplasia and cancer. Indeed, several studies have shown good prospective for E5 immunotherapy, suggesting that it could be applied for the treatment of pre-cancerous lesions. Thus, insofar as the majority of cervical, oropharyngeal and anal cancers are caused by high-risk HPV (hrHPV), mainly by HPV16, the aim of this review is to discuss the immune pathways interfered by E5 oncoprotein of hrHPV highlighting the various aspects of the potential immunotherapeutic approaches.

## Background

E5 is a small hydrophobic protein of Papillomavirus which is generally located in the endoplasmic reticulum (ER) and Golgi apparatus (GA), though it can also be found in the plasmatic and nuclear membrane surfaces [1]. Recently, E5 was proposed to be classified as a viroporin, a channel protein able to modulate ion homeostasis, vesicle trafficking, virion production and viral genome entry [2]. In HPV16 infected cells, E5 oncoprotein plays a key role in cell growth and impairs several signal transduction pathways. Furthermore, pro-carcinogenic activities are also performed by HPV16 E5, including the stimulation of EGF-mediated cell proliferation, the inhibition of apoptosis induced by Tumor Necrosis Factor Ligand (TNFL) and CD95 ligand (CD95L) [3] and the modulation of genes involved in cell adhesion and cell motility [4]. All of which are activities that indirectly intervene in the host’s immune system.

HPV16 E5 oncoprotein is also capable of interfering in the host’s immune response directly. It contributes to ensure HPV invisibility to the host’s immune system which is crucial for the onset and persistence of the infection and, consequently, for cell transformation and cancer progression. Therefore, immune cells cannot reach the infected sites or recognize the pathogenic agent, which keeps very low copies of itself; thus modulating innate and adaptive responses, inflammation and the synthesis of cytokines [5]. For that, HPV16 E5 oncoprotein carries out important anti-immune activities, such as the downregulation of classes I and II of the Major Histocompatibility Complex (MHC) on the cell surface, the intervention in the Cyclooxygenase-2 (COX-2) pathway and the prevention of natural killer (NK) cell and interferon activities. Despite the knowledge about E5, a great number of factors in determining the E5-related immune evasion mechanisms remain to be disclosed [6].

In general, the immune system is able to destroy the virus and eliminate the infected cells, but about 10% of infections can produce lesions that may undergo malignant transformation due to a failure of the immune response [7]. Due to this fact, immunotherapeutic methods appear as promising approaches to cervical and HPV-related cancers treatment. However, suitable medical therapeutic approaches have not yet been established, and an alternative route to achieve this goal could be the development of immunotherapies targeting E5, as suggested by recent studies [8–10]. In the following paragraphs, viral replication and transforming activity will be summarized emphasizing E5 contribution to those processes. Finally, the immune pathways affected by E5 and the various aspects of the potential immunotherapy approaches will be highlighted.

## Virus cycle

hrHPV is the main cause of cervical cancer and is also related to other tumors, such as head-and-neck [11] and anogenital [12]. It’s genome is composed of a Long Control Region (LCR) of nearly 1 kb and a region that encodes the early (E1, E2 and E4 to E8) and late proteins (L1-major capsid and L2-minor capsid proteins) [13].

Virus infection mainly occurs through sexual intercourse, generally by means of micro-injuries on cervical tissue. This event enables the virus to bind laminin-5 or -332 (a non-related heparan sulfate molecule) or heparan sulfate moieties, which may act as transient receptors in the extracellular matrix to attract the virus to the keratinocytes surface. Once the virus reaches the basement membrane of the epithelium, it interacts with several receptors for docking: first L1 and L2 bind the heparan sulfate proteoglycan (HSPG), which leads to conformational changes of these proteins; later, the attachment is to a non-HSPG molecule, whose identity has not been entirely elucidated yet. Then, the viral genome goes through the cell membrane by either calveolae or clathrin-dependent or -independent endocytic pathways, depending on the specific HPV genotype, and reaches the endosomal compartment [14].

Once internalized, HPV is uncoated and L1 dissociates from L2. L2 is suggested to be critical for HPV infection since it forms a complex with the viral genome and the nuclear domain 10, which are led together to the nucleus [14]. It is in this region that Papillomavirus initiates its replication and expression programs as an episome, and generates RNAs that undergo a large alternative splicing process. At this moment, the virus is into the basal layer, the expression of E1 and E2 genes is increased and a pool of infected cells with 10 to 50 copies of viral genome is formed. At this phase, there is no elimination of immunogenic particles that can be recognized by the immune system [13].

Once infected cells attain the parabasal and superficial layers, the expression of the other early proteins takes place. They keep keratinocytes in the proliferative stage, which prevents their normal differentiation process. In a final stage of virus cycle, novel virions are synthesized with the support of L1, L2, E2 and chaperone proteins [15]. Only now these particles can be released spontaneously along with the viral proteins, following the host cell natural apoptosis. This time is the suitable moment to ensure the nescience of the immune system, since the immune cells access is limited in the upper layers [5].

## The oncogenic proteins

The oncogenic viral proteins E5, E6 and E7, are able to modulate the expression of pivotal proteins that control mitogen activity, differentiation program and immune evasion mechanisms. As a result of E2 gene loss during viral genome integration, both E6 and E7 reach elevated expression levels, which are essential for cell transformation. They disrupt the normal cell capacity for apoptosis and replication and interfere with the S-phase entry. E6 is able to interact with several key proteins which are directly or indirectly involved in cell cycle regulation, gene expression, apoptosis and differentiation/mitogen balance, such as p53, E6AP ligase, p300/CBP, histone acetyltransferases (ADA3), AP-1, Bak, Bax, FADD, procaspase 8, ERC-55 and paxillin [16]. E7, in turn, is capable of interacting with pRb, cyclin A/CDK2 (cyclin-dependent kinase 2), cyclin E/CDK2 (indirectly), p27 and p21 [17].

The transforming activities of E6 and E7 are supported by E5, which also has its own oncogenic properties. E5 induces mitogenesis in several ways and regulates important growth factor receptors or molecules, such as the epithelial growth factor receptor (EGF-R), Bcl-2, Bax, Fas and calnexin, which are involved in the control of cell differentiation, survival and growth [3, 5].

However, different HPV types express distinct forms of E5 and this causes differences in the carcinogenic competence of the Papillomavirus. Indeed, low-risk HPVs (lrHPVs) lack E5 or encode different polymorphic types with less transforming ability (E5β, -γ, -δ), resulting in non-tumoral clinical outcomes, whereas hrHPVs encode another E5 form (E5α) that may lead to lesion progression and tumor development [18]. It is known that different forms of E5 have different abilities of binding the EVER and ZnT-1 proteins (these proteins are important in viral genome replication and immune response). HPV16 E5 (E5α) is able to bind and prevent the activities of EVER1, EVER2 and ZnT-1, which results in the inhibition of MTF-1 transcriptional effect. Hence, the different cell transformation ability of E5 from high- and low-risk HPVs could be directly related to this feature, insofar as the other types of E5 (β, γ and δ) are not able to bind these proteins [19]. Thus, this finding indicates that the difference between hrHPVs and lrHPVs could be determined by the presence of a particular E5 ORF in ELR region (region between the early and late genes of HPV genome). Therefore, the oncogenic competence of HPV could be extensively related to E5 oncoprotein and this subject is worthy of further experimental studies [18, 20].

It is well established that HPV16 E5 oncoprotein acts in the early stages of infection/transformation [5] and, in particular, in DNA synthesis in suprabasal epithelium and in viral genome amplification along with the E7 oncoprotein [21]. After the viral DNA is integrated into the host’s genome, E5 gene is usually lost, suggesting that its activity is not required for the late transformation stage. However, some studies demonstrated that HPV16 E5 remains expressed even in high grade lesions and invasive cancer. This might be due to the presence of an episomal form or head-to-tail integration, which is responsible for E5 expression [22]. In unpublished data, our group confirmed the HPV16 E5 mRNA expression in biopsies from patients with high grade and cancerous lesions. Thus, it is likely that this oncoprotein may interfere with all stages of tumorigenesis.

Finally, since E5 is mainly active in the early phase of infection, it has become a key target of therapeutic studies which seek to prevent the progression of the lesion to pre-malignant stages and carcinoma [23].

## The host immune response

The host’s immune system harbors both innate and adaptive immune responses, which are responsible for a successful protection against the papillomavirus and tumor development [24]. However, HPV can sometimes escape from all immunologic efforts, due to its oncoproteins activities, including E5. In the following paragraphs, it is presented how some key agents from the host’s immune system act in a non-infection condition and, in the following topic the E5 activity on these agents is discussed.

The initial barrier to genital infection by HPV is the cervical epithelium that contains keratinocytes (KC) and dendritic cells (DC), both of which are responsible for antigen presentation to T cells through MHC I and II. Keratinocytes are able to induce DC and NK cell maturation and to promote CD4^+^ and CD8^+^ T cells activity. Similar to KC, DC recognizes antigens through the pattern recognition receptors (PRRs) and synthesizes a wide range of signaling molecules, including interferons and cytokines. This cell is also capable of direct interaction with the NK cell, promoting its activation. These initial innate immune response efforts are essential to the development of an effective clearance of HPV, but in a minority of occasions, E5 and the other oncogenes are able to successfully escape from host immune surveillance [25, 26].

In the next sequence of events, the activation of T cells comprises the major effector cells for the regression and eradication of HPV infection. These cells have cytolytic capacity and synthesize antigen-specific antibodies and a wide range of signalling mediators in order to maintain antigen-specific memory B cells and lyse infected/tumour cells. They establish a specific cytokine profile concerning the antitumor response, in a complex balance between Th1, Th2, Th17 and Treg cytokines which won’t always result in cancer clearance. The absence of T cells were associated with persistent infection and neoplastic progression [5, 25] and, because of that, the priming of these cells is continuously used as immunotherapeutic targets [27, 28].

In this scenario, the presence of checkpoint molecules/receptors and their inhibitors also play an essential role supporting cancer development. As an example, the receptors KIR, CTLA-4 (Cytotoxic T-lymphocyte antigen-4) and PD-1, and its ligand, PD-L1, are immunosuppressive molecules, currently associated with a poor prognosis [29, 30].

The identification of CTLA-4, a protein receptor on the surface of T cells, dates back to the late 1980s, when it was shown that it puts brakes on T cells, preventing them from launching full-out immune attacks and reducing them to a small pool of memory cells [31]. As well as CTLA-4, PD-1 is also present in T cells and activates an inhibitory cascade, hampering T cell responsiveness and its cytokines secretion. Its ligand, PDL-1, which is expressed in antigen presenting cells (APCs), was also found substantially expressed in tumour cells of HPV-related cancers [32–34]. However, a complete description of this important immunosuppressive milieu goes beyond the scope of this review and many reviews dealing with this issue have been recently published [29, 30, 35].

## Immune modulation by E5 oncoprotein

The major immune mechanism disrupted by E5 oncoprotein is antigen presentation, accomplished by MHC antigen processing. Following the guiding principles of the “missing-self” hypothesis, other viruses, as HPV, disrupt MHC expression in a variant-selective manner to protect infected cells against the NK and CTL cytotoxicity. This occurs by a selective downregulation: while HLA-C and the non-classical MHC class I molecule HLA-E, which binds to the inhibitors receptors of NK cells, maintain their constant levels, HLA-A and -B are downregulated and are not able to induce CTL activation and accomplish infected cell lysis [36, 37].

Classical MHC is classified in class I (HLA-A, -B and -C genes) and class II (HLA-DR, -DQ, -DP genes). MHC I is a complex formed by a polymorphic heavy chain codified by the loci HLA-A, -B and -C in chromosome 6, and by a light chain, denominated β2-microglobulin, codified in chromosome 15 [38]. These HLA types are responsible for coupling antigen peptides in ER, which are transported to GA and shown at the cell surface to T cytotoxic lymphocytes [39]. In infected cells, however, HPV16 E5 blocks this mechanism. It is of common knowledge that MHC I is downregulated at the membrane surface of keratinocytes and, thus, the viral antigen recognition and maturation of NK and T cells are impaired. This effect is linked to the interaction between the hydrophobic transmembrane domain of HPV16 E5 and the heavy chain of MHC I in Golgi/ER membrane [40] and not by the blockage of the expression of the heavy chain or the transporter protein TAP1 [36].

It was also shown that HPV16 E5 can downregulate the MHC I membrane expression, by causing alkalization inside the membrane compartments, which keeps MHC I inside the GA [6], and by interaction with the chaperone calnexin, which causes retention of MHC I inside the ER [41]. A similar mechanism to this occurs for CD1d, a MHC I-like glycoprotein that presents self or microbial lipid antigen to NKT (natural killer T) cell. The downregulation of this molecule, which will eventually be degraded at the cytosol, is an immune evasion strategy performed by several other viruses besides HPV. CD1d is important for antigen presentation to CD1d-restricted invariant NKT cell (iNKT) that, once activated, plays a key role in anti-viral response since it causes the lysis of infected cells and modulates Th1/Th2 polarization [42].

In vitro studies showed that the HPV16 E5 interaction with the 16 kDa subunit of ATPase resulted in decreased MHC I levels and a reduction of CLT cells recognition and activity [5]. The same results were found in a bovine model. This mechanism of altered transport was further evidenced by the lack of increased surface levels of MHC I even when total levels of MHC I were increased by interferon (beta and gamma) treatment [43]. Similarly, HPV16 E5 was able to interfere with MHC II surface expression [44].

**Fig. 1:**
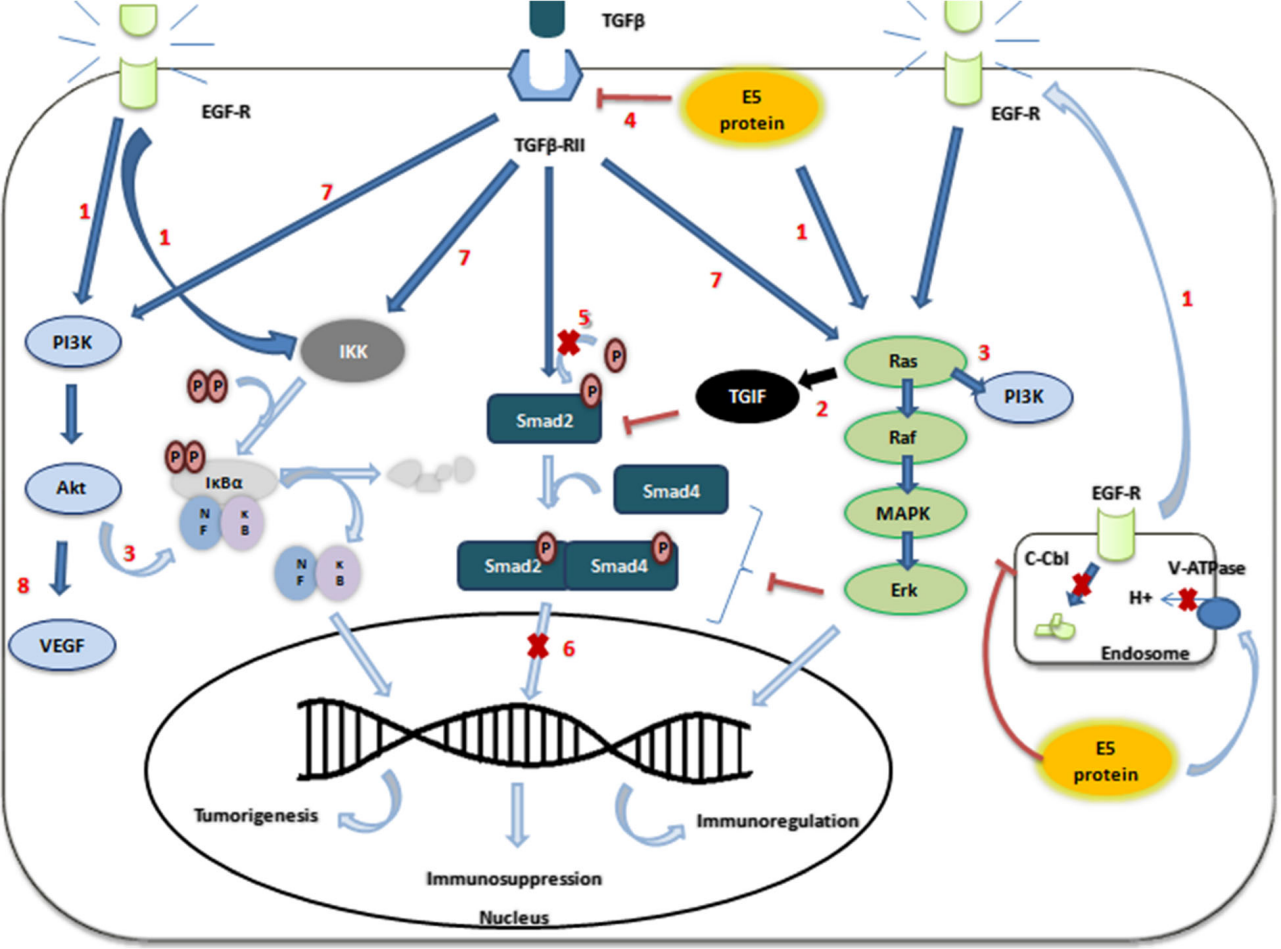
EGF-R and TGF-β signaling pathways interfered by E5. EGF-R and TGF-β share several signal transduction pathways, so that the inteference of hrHPV E5 on any of these receptors have effects on each other. TGF-β is an important immunosuppressive cytokine, which activates different transduction pathways interposed by E5 activity. In a natural condition (without any infection), TGF-β prioritizes SMAD pathway, which results in expression of several tumor suppressive proteins. In the HPV infection, SMAD pathway is hampered by HPV oncoproteins, whereas other alternative pathways are stimulated. (1) E5 is capable of increasing the EGF-R levels (by preventing the activities of c-Cbl and V-ATPase) and activating MAPK-ERK, NF-κB and PI3K pathways. (2 and 3) These pathways interact each other in an intricate regulation way. Ras protein stabilizes TGIF [75], a co-inhibitor of SMAD pathway. Moreover, ERK activation inhibits the SMAD activity through phosphorylation [77] and activates PI3K and NF-κB pathways [78]. In turn, activation of NF-κB and PI3K pathways by EGF-R cause a negative feedback on SMAD pathway. (4) E5 also inhibits TGF-βRII expression, (5) SMAD2 phosphorylation and (6) SMAD2-SMAD4 complex translocation to the nucleus [68]. (7) The activation of non-SMAD pathways leads to cell proliferation and disruption of cytokines synthesis, which stimulate tumor progression. (8) E5 can also can stimulates VEGF through EGF-R-PI3K-Akt signaling inducing angiogenesis [144]

Besides interference with antigen presentation, HPV16 E5 also modulates the immune and inflammatory pathways through other manners by interfering in EGF-R activation and transduction signaling such as the mitogen-activated protein kinases (Ras/Raf/MAP kinase) and the phophoinositide 3-kinase (PI3K/Akt) [6]. The first transduction pathway is involved in cytokines synthesis [45] while the second regulates chemokine production, DC differentiation from monocytes, NK maturation and leukocytes migration [46]. EGF-R activation also induces an increase in the expression of ganglioside-1 (GM-1) and caveolin-1 on the cell surface, affecting cell signaling and vesicular trafficking. GM-1 inhibits cytotoxic T lymphocytes and immune synapse formation, and increases the EGF-R proliferative response [47]. Figure 1 summarizes several other transduction molecules which are involved in EGF-R signaling, including the ones exemplified above, besides the E5 interferences in TGF-β signaling mediators discussed in "Transforming growth factor β signaling" topic.

**Fig. 2:**
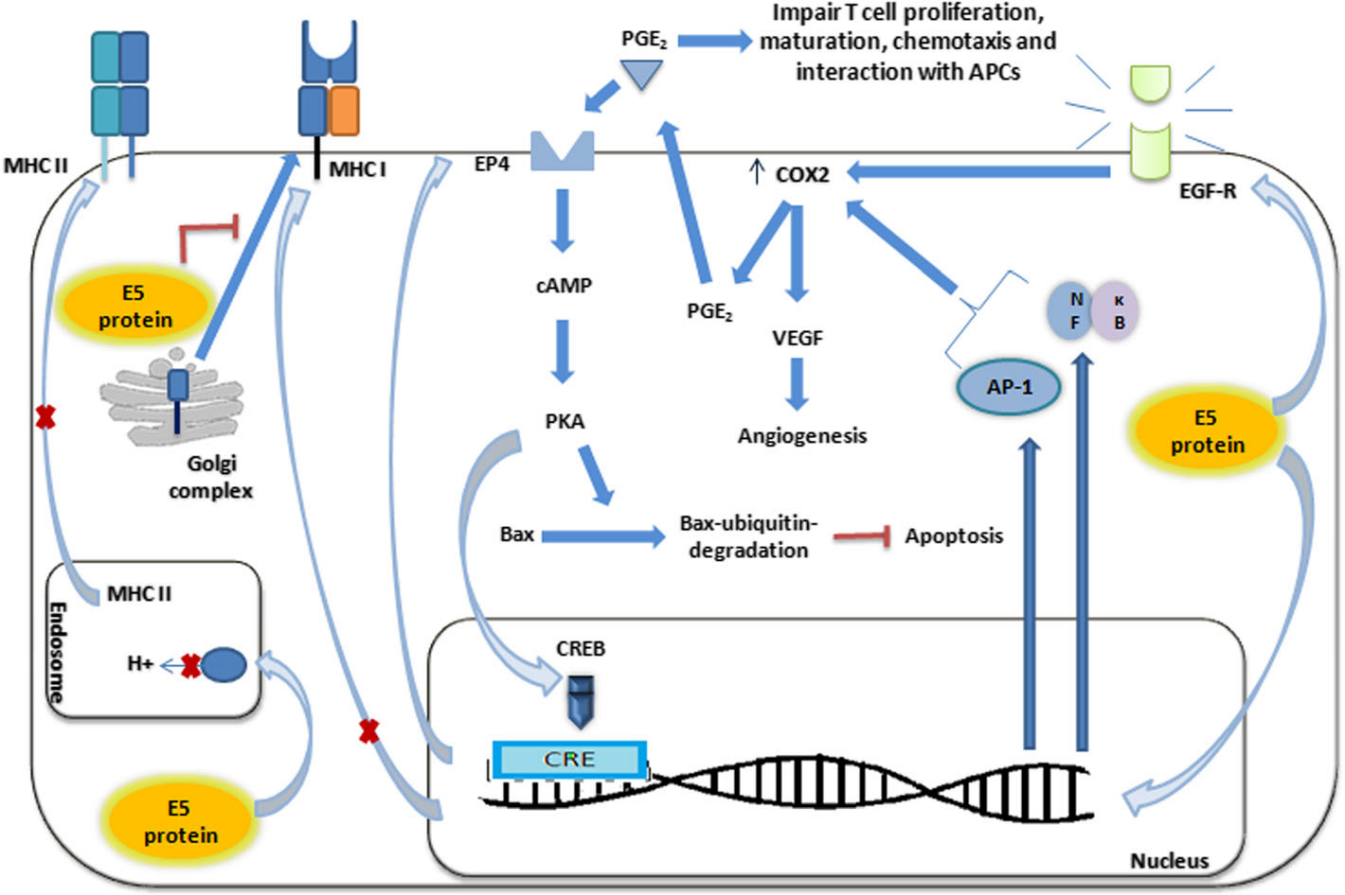
E5 immune evasion mechanisms. E5 induces COX-2 expression through: i) the activation of the epidermal growth factor receptor (EGF-R) signalling pathway and the interaction with the nuclear factor 1 (NF-1), which results in the induction of ii) AP-1 and iii) NF-κB transcription, which has the strongest effect on COX-2 transcription. E5 upregulation of EGF-R expression also leads to an increase of the vascular endothelial growth factor (VEGF), through COX-2 [48]. Following this, COX-2 and EP4 stimulate PGE2 signalling pathway through a feedback mechanism involving increased levels of cAMP, PKA and CREB (cyclic adenosine monophosphate response element binding protein), binding to the EP4 promoter [53]. This leads to a rise in the expression of this receptor [87, 88], which is associated with breast and colon carcinogenesis [145, 146]. Moreover, E5 impairs MHC I [36] and II [44] surface cell expression

EGF-R activation by HPV16 E5 also interferes in the inflammatory pathway. After EGF-R phosphorylation, this receptor is able to induce the transcription of COX-2, whose upregulation was usually associated with malignant processes and with a decreased overall survival rate and increased metastasis [48, 49]. The kappa B nuclear factor (NF-κB), as well as the prostaglandin E2 (PGE_2_), both related to COX-2 pathway, were also proposed as bad prognostic factors. NF-κB is a key factor of immune response [50, 51] and PGE_2_ seems to be involved in anti-inflammatory response, since it impairs T cell arrest and interacts with APCs, which compromises T cell maturation [52]. In addition, this prostaglandin stimulates cellular proliferation, angiogenesis, cell migration, invasion and survival [53] (COX-2 pathway can be seen in details in Fig. 2). Therefore, both molecules may be an alternative target for therapeutic interventions, since recent trials have shown that the inhibition of COX-2 led to adverse side effects [49].

In addition, E5 is responsible for the deregulation of other important key participants in the immune response, such as the natural killer cells, the transforming growth factor-β and interferons, as highlighted in the following sections. Table 1 summarizes the E5 influence on host immune activities.

**Table 1:** The influence of the HPV16E5 oncoprotein on immune system

E5 activity	Mechanism
Disruption of the transport of MHC I to the surface, reduction of antigen presentation to CTL cells and NK-mediated response.	E5 prevents the transport of MHC I (HLA-A and –B) to the surface membrane in three significant ways: i) it impairs Golgi Apparatus acidification which causes the accumulation of MHC I in this organelle [5]; ii) it binds to Bap31 (B-cell-associated protein 31), by displacing this protein from MHC I and causing the retention of this molecule in ER/GA [136]; iii) and it interacts with the MHC I heavy chain, via leucine pairs [5].
MHC I and MHC II downregulation.	By impairing MHC I [36] and II [44] gene expression, E5 supports HPV immune evasion.
Inhibition of CD1d-mediated activities.	E5 binds to calnexin and thus traps the CD1d molecule into the endoplasmic reticulum, reducing CD1d levels at the membrane surface [42, 137].
Increase of EGF-R availability causing an upregulation of COX2, VEGF and inducing cell proliferation.	E5 binds and inhibits the activity of V-ATPase, impairs endosomal acidification and causes the degradation of EGF-R. It also enhances EGF-R recycling at the plasma membrane [138, 139]; it also delays EGF-R degradation owing to interference with membrane trafficking [3] and interaction with c-Cbl, provoking decrease of c-Cbl-mediated degradation of EGF-R [140]. These activities cause upregulation of COX2 [49], which is an essential enzyme for inflammatory response, and VEGF, an angiogenic factor [141].
EGF-R-dependent or -independent activation of signaling pathways.	E5 activates MAPK, p38 and extracellular signal-regulated protein kinases 1 and 2 (ERK1/2) independently of EGF-R activation, which increases the expression of *c-fos* and *c-jun* (types of AP-1) and stimulates the transcription of E6 and E7 oncogenes [6, 23, 142]. Besides, it impedes several host immune protective activities. E5 can also bind directly to EGF-R and other growth factor receptors by hydrophobic interactions and induce the ligand-dependent signaling of these receptors [143].
Activation of gene expression of caveolin-1 and ganglioside-1.	Caveolin-1 and GM-1 are upregulated in the plasma membrane, and support viral immune evasion [47].
Upregulation of IFN-β.	IFN-β gene expression is induced by E5 by inducing the increase of IFN regulatory factor-1 (IRF-1) levels in infected cells [90].
Down-regulation of TGF-β-RII gene expression and TGF-β\SMAD signaling.	E5 attenuates TGF-β/SMAD-signalling by preventing TGF-β-RII gene expression. It also reduces SMAD phosphorylation and nuclear translocation [68].

### Natural killer cells

An important participant in the innate immune response is the NK cell, a subset of lymphocytes lineage with CD56^+^, CD16^+^, CD69^+^_,_ CD3^-^ and NKp46^+^ markers. This cell has a close relationship with DC and together they modulate effector responses against HPV. They support the lysis of infected/tumour cells and secrete various inflammatory cytokines/chemokines, which support the maturation and priming of T cells and the development of a viral antigen-specific response, including interferon-γ, TNF-α, MIP-1α (macrophage inflammatory protein-1α), GM-CSF (granulocyte-macrophage colony-stimulating factor) and others [54].

The NK cell is capable of attacking targeted cells without previous antigen exposure by the recognition of non-self and self HLA and a delicate balance between receptor signalling – positive (i.e. NKG2D, NKp30, NKp44, NKp46) and negative (i.e. NKG2A). Previous studies demonstrated that cessation of NK cell activity led to tumour growth and metastasis [54], so it is considered a poor prognostic marker for HPV-related cancer. hrHPVs are able to reduce these cells’ levels and downregulate the expression of NK-activating receptors at cell surface [55–57]. These events lead to a successful evasion of the first line of defence accomplished by NK cells.

The pathway activated by NKG2D receptor can positively modulate NK cells activities and it seems to play an important role in cancer studies. This receptor is located on NK cells and is capable of interacting with classical MHC I-related molecules MICA, MICB and ULBPs (UL16-binding proteins) and inducing NK cell anti-viral defence mechanism [54]. However, it was also observed that these ligands may cause NKG2D down-regulation on NK cells, which suggests the existence of a feedback control mechanism [58]. Furthermore, cervical cancer showed a low expression pattern of NKG2D in NK cells along with the reduction of NK cells cytotoxicity [55]. Thus, it is possible to speculate that since E5 exerts an extensive activity against several MHC molecules, it may disrupt the processing and maturation of these ligands (MHC I-related molecule) since they have a MHC-like structure.

Additionally, E5 possibly affects NK cell activation by the modulation of Treg cells and TGF-β. Treg cells act primarily through the secretion of antitumor cytokines, including TGF-β, which was found upregulated in hrHPV infection. It was reported that TGF-β was able to disrupt NK and T cell activities by inducing the expression of inhibitory receptors and inhibiting the stimulatory ones [26, 59, 60]. This factor also induced NK cell lysis and the downregulation of NKG2D. As a result, a feedback loop consisting of E5 (hrHPV), Treg and NK cells, is able to potentialize the induction of immune tolerance, which creates a favorable environment for tumor’s establishment and progression. In a physiological situation, though, NK cells are capable of suppressing Treg cell maturation and promoting infected cell lysis in a NKG2D-dependent manner [61].

Furthermore, TGF-β can modulate NK cells by inducing the release of soluble MICA (sMICA) in renal epithelial cells [62] and head-and-neck squamous cell carcinoma [63], which downregulates NKG2D. The TGF-β/MICA/NKG2D pathway was also found altered in other tumors [64–66] and viral infections [67], suggesting that the deregulation of this pathway is relatively common and important in cancer immunology. Likewise, this immune evasion mechanism was found to exist in HPV benign infections [64], probably by HPV16 E5 modulation [68]. However, no studies have accessed the importance of this pathway in the malignant progression of cervical carcinogenesis. The only tested factors were the sMICA and NKG2D in the serum of patients with cervical cancer and precursor lesions without measuring TGF- β and/or E5 expression levels [64].

### Transforming growth factor β signaling

Epithelial and Treg cells produce TGF-β, which is involved in a wide range of cell mechanisms, including immunity, angiogenesis, cell proliferation, apoptosis and inflammation. This factor operates by interacting with two cell surface receptors, the TGF-β receptors (TGF-βR) I and II, which trigger signal transduction primarily through the SMAD protein phosphorylation [68].

The large TGF-β family includes several different cytokine types, including TGF-β1, TGF-β2 and TGF-β3. They bind to the constitutively activated TGF-βRII that, in turn, activates TGF-βRI and initiates receptor-associated SMAD (R-SMADs) phosphorylation. Once it is phosphorylated, R-SMADs forms a complex with co-operating SMAD (co-SMADs) and both proteins are translocated to the nucleus, where they induce gene transcription through DNA binding and interaction with transcriptional factors. The TGF-β family can also act through alternative signaling pathways like MAPK, NF-κB, Rho-like GTPase, PI3K/Akt and PP2A/p70s6K (Fig. 1) [69]. The modulation of these other pathways by TGF-β is always cell type- and condition-related [70].

In physiological condition, TGF-β1 acts as a tumor suppressor by promoting apoptosis and cell cycle arrest. In contrast, mutations and deletions in TGF-β/SMADs signaling cause a switch in TGF-β response, from inhibition of cell growth to promotion of tumor proliferation, migration and invasion [69]. The expression of this factor and its activity are also altered during tumor progression [71]. Indeed, TGF-β expression levels were found altered by HPV presence and decreased as cell malignancy progress from cervical intraepithelial neoplasia to carcinoma [72], being suggested that it owns a dual role: it acts as a growth inhibitor in low-grade lesions, but as pro-oncogenic in high-grade and carcinoma lesions [73].

By the activation of the SMAD pathway signaling, TGF-β1 has an important immunomodulatory effect, which promotes self-tolerance and prevents deregulated cell proliferation. This cytokine is responsible for modulating the T cell differentiation, promoting a shift from Th1 to the Th2, Th17 and Treg profiles, and it also blocks T CD8^+^ proliferation and differentiation, NK cell cytotoxicity and DC activities [74].

In cervical cancer, HPV16 E5 induces the downregulation of TGF-β/Smad signaling, an extremely important tumor suppressor route [71]. An in vitro and in vivo study showed that an increased HPV16 E5 expression led to reduced expression of TGF-βRII [68]. This event might be the trigger for TGF-β to allow a shutdown of the tumor suppressor role, since the activation levels of TGF-βRII are essential to achieve a TGF-β anti-tumor outcome [71]. Supplementary, it was also found that Smad2 (R-SMAD) phosphorylation and Smad2/Smad4 (R-SMAD/co-SMAD complex) nuclear translocation were clearly reduced in HPV 16 E5-positive cells [68].

In addition to the alteration of TGF-βR/SMAD pathway, HPV16 E5 was able to stimulate the EGF-R/MAPK/Ras pathway. In this particular route, the activation of the Ras protein stabilizes the SMAD co-repressor TGIF (homeodomain protein TG-interacting factor), causing failure in the SMAD signaling transduction [75] and inducing Smad4 degradation [76]. Furthermore, MAPK/ERK activation results in inhibition of the SMAD activity through phosphorylation of specific sites [77] and in activation of other alternative pathways such as PI3K and NF-κB [78]. The transduction pathways are concurrent and have reciprocal negative interactions in the keratinocytes. The alternative MAPK/Ras pathway is generally associated with the occurrence of several cancers [79, 80] and with NF-kB-mediated tumorigenesis [45].

In summary, tumor progression requires a balanced regulation between mitogenic stimulation and immune evasion. E5 seems to play a role in carcinogenesis through the prevention of TGF-β/SMAD signaling which stimulates cancer formation, and indirectly leads to an increase of TGF-β through immunosuppressive Treg upregulation. Since this growth factor is a mediator of several cellular pathways and different immune responses, its alterations can greatly disturb cell homeostasis, and thus may provide a useful potential tool for therapy of pre-tumoral lesions and cancer.

### Interferon pathway

Interferons (IFNs) are key signaling mediators for generation of an antiviral state and infection clearance, which are synthesized by T lymphocytes [81] and NK cells [82]. They are classified as type I, II and III [83] and their transcription occurs by the modulation of important signal transduction pathways (i.e. NF-κB) and molecules (i.e. IRFs, AP-1 etc) [84] which bind to specific sequences on DNA called ISRE (IFN-stimulated response elements) [85]. The reduction of their expression levels are associated with malignant transformation and tumor progression [83].

**Fig. 3:**
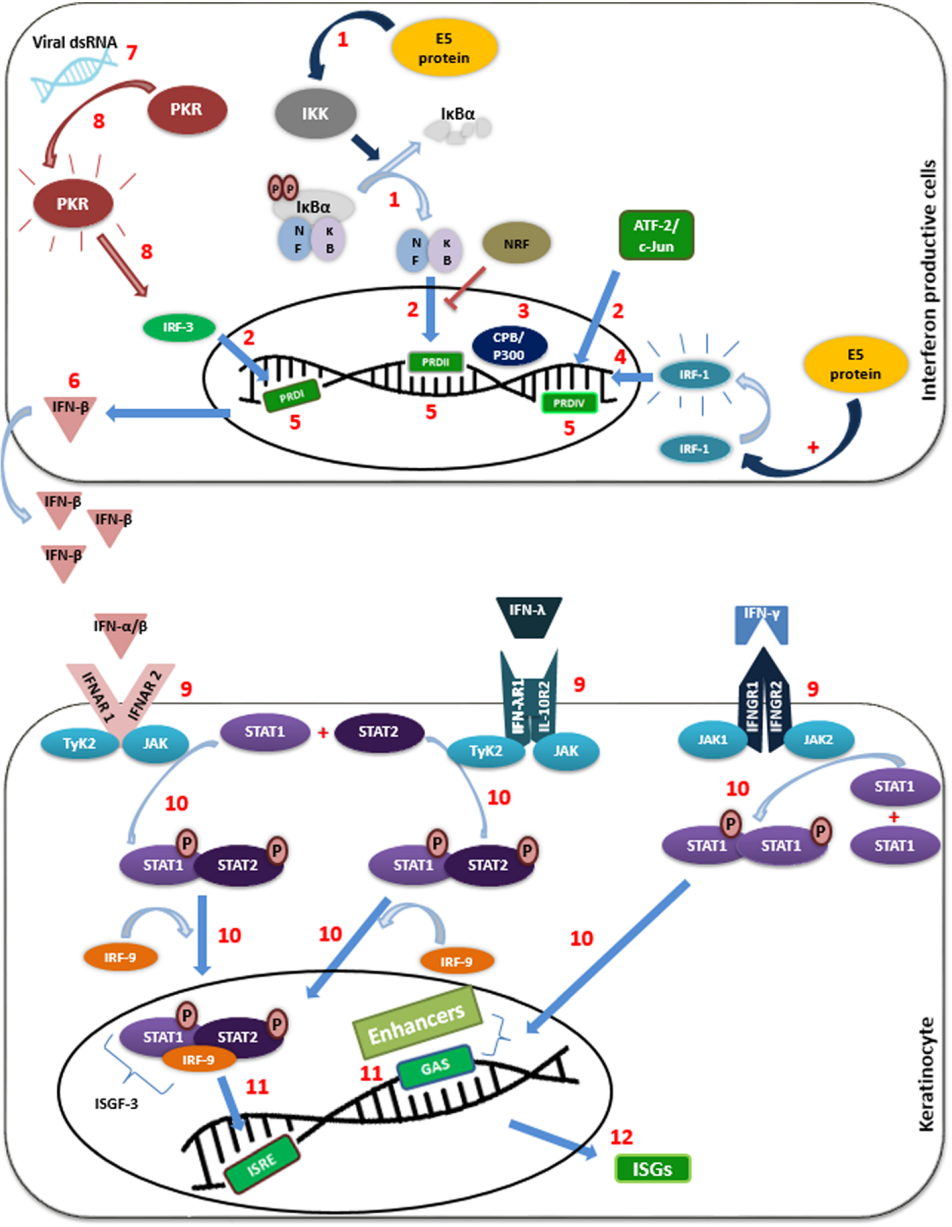
Interferon synthesis and signaling pathways. Interferons are crucial molecules for creating antiviral status. E5 stimulates IFN synthesis through activation of NF-κB signalling pathway and IRF-1 protein. (1) PRR/MAVS (Mitochondrial antiviral signaling) activates IKK that liberates NF-κB. (2) NF-κB, IRF-3 and ATF-2/c-Jun form a transcriptional complex that recruits the (3) CPB/P300 enhancer to IFN-β promoter. (4) This complex (along with IRF-1) binds to (5) particular DNA regions (PRDI, PRDII and PRDIV) which results in (6) IFN-β gene transcription. In IFN-independent way, (7) viral dsRNA induces (8) PKR-IRF3 signalling. The produced IFNs exert their activities in keratinocytes by (9) interacting with specific IFN-type receptor and (10) triggering JAK/STAT signalling pathways. IFN-α, IFN-β and IFN-λ interact with receptors associated with JAK1 and Tyk2 tyrosine kinases which induce the activation and dimer formation of the transcriptional factor STAT1/STAT2 by phosphorylation. This dimer forms a complex with IFN-stimulatory gene factor- 3γ (ISGF-3γ), also called IFN regulatory factor-9 (IRF-9) or P48, which bind to the ISRE sequence of DNA. In turn, IFN-γ binds to receptors associated with Jak1 and Jak2 tyrosine kinases that induce the formation of the Stat1/Stat1 homodimer. Finally, (11) the binding of transcription factors to specific responsive elements (i.e. ISRE and GAS) of DNA leads to (12) ISGs expression [99]. This IFN-induced activities create an antiviral state which leads to the destruction of infected cells with episomal HPV [90], whereas cells with integrated viral DNA can survive and transcription of E6-E7 oncogenes are no longer regulated by E2 leading to persistent infection and cancer formation [90]

Type I IFN has a potent anti-viral systemic response and includes more than 20 members, including IFN-α and IFN-β. Type II IFN (IFN-γ), in turn, plays a key role by establishing a bridge between innate and adaptive immune responses by promoting cytotoxic response and T cell activation. Finally, the most recently discovered type III IFN family seems to have a similar anti-viral and anti-tumor properties to type I IFN, but without a systemic activity [86]. Thus IFNs class III are able to activate several immune cells, such as DCs, NK cells and macrophages, as well as to inhibit neutrophil recruitment and Th1 and Th17 responses [87]. The three classes of IFNs act via JAK/STAT signal transduction pathway with small differences (interferons signal transduction pathways can be seen in details in Fig. 3).

In the absence of viral infection, keratinocytes normally express low levels of IFN and the same happens when an hrHPV infection occurs, which leads to a failed immune response [88]. Keratinocytes infected by HPV should trigger an efficient type I IFN response that has antiviral and anti-tumor properties, so that innate and adaptive immunity can be activated. Instead, as other DNA virus, hrHPVs developed the ability to inhibit IFN I activity [89]. In clinical samples, type III IFNs as well as ISGs (interferon stimulated genes) responses were activated in lrHPV infection, whereas they were downregulated in hrHPV infection [88].

In HPV16 infection, it was observed that E5 is able to regulate IFN synthesis and signaling pathways in order to support carcinogenesis. It upregulates IFN-β through the activation of IRF-1, which binds to the IFN-β promoter and induces its transcription [90]. This establishes an antiviral state, but does not impair disease progression; it leads to the onset of the infection and carcinogenesis. The lesion progression can be attributed to a change in the physical status of the viral DNA in the infected tissue, with an IFN-induced loss of episomal HPV in infected cells and persistence of cells containing an integrated form of HPV in the early stage of infection [91].

The powerful anti-viral and anti-tumor effects of IFNs have been evaluated in several clinical studies assessing their efficacy as therapy for autoimmune diseases [92], viral infection, and cervical cancer. Promising results have been achieved with IFN treatment in association with a vaccination against HPV16, consisting of E6-E7 synthetic peptides [93, 94]. IFNs are also capable of inducing apoptosis of infected cells and stimulating an antiviral state in healthy cells [95]. Thus, the correct use of interferon in therapy may depend on the selection of the correct time and the correct conditions for their application. Type I IFN might have good prospects of therapeutic efficacy in cells with episomal HPV where it leads to apoptosis and cell growth arrest, but cells with integrated HPV genomes are far less sensitive [90]. This fact may explain the controversial results that were recorded about interferon therapy in low-grade lesions and hrHPV-induced cancer and the successful treatment of lrHPV-induced genital warts [96, 97]. Moreover, IFN therapy of CIN lesions led to inconclusive results hampering a wide application of this treatment [98]. Although type I IFN was used as a potential antiviral therapy, some data indicates that type II IFN (IFN-γ) is more effective in HPV infected cells [99].

Finally, type III IFN seems to be a very promising therapeutic tool because its compartmentalized immune response attacks viruses in epithelial tissues avoiding the systemic side effects of inflammation [100]. Unfortunately, to the best of our knowledge, no therapeutic evaluation of type III IFN and E5 was made, but its connection with several types of cancer, including cervical, were established [101]. HPV infection activated type III IFN in cervical HPV-positive biopsies and hrHPV infected tissues showed decreased levels of this IFN, suggesting that the lack of this type of IFN may be related to lesion progression [88].

## Immunotherapy

The study of the interaction between HPV and the host’s immune system has opened up a number of therapeutic opportunities [102, 103]. These therapeutic strategies aim to modulate immune responses for treating or preventing infection. Since there are no antiviral drugs for HPV infection, cervical lesions are treated by chemotherapy, radiotherapy, surgery or hysterectomy as the most radical of all. However, these procedures are not effective in all cases, which results in a high recurrence and significant global mortality rate, around 52% or more than 270.000 deaths per year [104]. When HPV-related cancers are included, the number of deaths raises to 295.000 per year or one every two minutes [105]. About 95% of anal, 70% of oropharyngeal and 65%, 50% and 35% of vaginal, vulvar and penile cancers, respectively, are caused by hrHPV [106].

Two prophylactic vaccines (bivalent and quadrivalent) were produced to prevent HPV infection [107], and recently a 9-valent vaccine was approved, which protects from nine HPV genotypes (6, 11, 16, 18, 31, 33, 45, 52, and 58) [108]. However, there is still a lack of therapeutic vaccines. These vaccines usually aim to activate immune cells, either from innate or adaptive response [109], in order to simulate the natural immune response and reverse the viral immunomodulatory mechanisms. It was reported, for example, that T CD4^+^ and CD8^+^ cells levels were reduced in high-grade cervical lesions [110], unlike what happens in spontaneous regression lesions [111]. Beyond that, a specific cytokine expression profile was also described in these opposite situations (lesion progression and lesion remission) [112, 113] and all these mechanisms allowed the adoption of a number of immunotherapeutic approaches of great potential, including those related to E5. Nowadays, the use of new immunotherapeutic approaches have emphasized the strengthening of non-specific immune response (i.e. cell-mediated responses, cytokines production and secretion) and stimulated researches about the synthesis of monoclonal antibodies against checkpoint inhibitors such as PD-1/PDL-1 and CTLA-4 [114].

HPV oncoproteins have been widely employed in cervical cancer immunotherapy and E6 and E7 were extensively tested. However, these two oncoproteins were not able to fully eradicate pre-cancerous lesions and for this reason, E5 has emerged as a third alternative [9]. The use of HPV E5 protein in therapeutic vaccines does not aim to eradicate E5 function, but to stimulate the host’s immune system to combat viral infection and lesion progression. It is well established, for example, that dendritic cells play a key role in the development of innate and adaptive responses due to its antigen presentation activity [115] and E5 can impair this activity. In addition, circulating DCs are reduced during viral-induced cell transformation and cervical cancer progression [116]. Therefore, an autologous DC administration, previously pulsed with E5 antigen in culture, could be performed to bypass this problem and stimulate properly the host’s immune system against virally infected cells.

So far, several types of therapeutic vaccines against HPV16 were analyzed: peptide-based, protein-based, DNA/RNA, viral/bacterial vector, plant-derived and DC-based vaccines. All of those methodologies were applied for E6 or E7 vaccines but only three of them (peptide-based, DNA, and viral vector preparations) were utilized for E5 vaccines. Among the methodology tested for E5, viral vector vaccines are a promising therapeutic strategy for the presentation of pathogen antigens to sensitize the host’s immune system, inducing a strong cytotoxic response. These vaccines are highly immunogenic since they are able to go inside the host cells where they synthesize the antigens of interest and cause the lysis of infected cells [109].

One of the first studies to evaluate HPV16 E5 therapeutic vaccine utilized recombinant adenovirus bioengineering to express E5 in a murine model. This vaccine produced a reduction of tumor growth in vitro and the immunological response was T CD8^+^ dependent and T CD4^+^-independent, suggesting that HPV16 E5 is an antigen able to induce tumor rejection [117]. In another study, a recombinant vaccinia virus for Bovine papillomavirus 1 (BPV-1) [118] E5 was effective in reducing experimental mouse tumors, but the same technology applied to HPV16 E5 elicited no positive immune effect, unlike HPV16 E6 and E7 [119]. In a DNA vaccine encoding HPV16 oncoproteins, E5 was associated with E6 and E7 and genetically fused to herpes virus glycoprotein D. This vaccine showed a specific and substantial T CD8^+^ response for each oncoprotein individually in a TC-1 tumor mouse model. This vaccine exhibited a higher anti-cancer (to 100%) effect when vectors encoding GM-CSF or IL-12 were co-administrated [120]. These cytokines are co-stimulators of immune response and activate/recruit central cells of innate system such as dendritic and NK cells [121], which results in maturation of APC and CTL response. Both co-stimulators were successfully utilized in pre-clinical and clinical trials of cervical cancer therapy, in particular IL-12 [103, 121, 122]. This strategy was utilized with some success by Borysiewicz et al. in the first human trial of immunotherapy with E6 and E7 recombinant vaccinia virus more than 20 years ago [123].

When comparing the results, a bias could be the different viral vector chosen. Unlike adenovirus, which was capable of inducing a CTL response for different recombinant virus, the vaccinia virus was unable to stimulate an appropriate immune response against HPV16 E5. It is known that adenovirus is capable of inducing a CTL response for different recombinant virus. Moreover, different tumor cells were used to induce tumors in mice model, which makes it difficult to compare the results. However, these studies suggested that HPV16 E5 is able to stimulate a host immune response with the induction of E5-specific cytotoxic T lymphocytes. These cells were activated by dendritic cells, thus, receptor agonists can further strengthen their stimulation to achieve a successful therapeutic outcome.

Additionally, the T cell-mediated immune response induced by HPV16 E5-peptide (delivered through recombinant adenoviruses) may be influenced by specific HLA-A, in terms of intensity-response. The HPV16 E5 epitope reduced the oncogenic potential and generated specific T memory cells that are restricted to HLA-A*0201, observed in patients samples, unlike the whole HPV16 E5 oncoprotein, which did not show signs of this ability [124].

Potent peptides can be used to induce a host immune response against viruses [93, 109] and HPV16 E5 showed *in silico* evidence of being an useful therapeutic strategy [125]. Peptide-based vaccine is a safe and easy methodology to execute despite having a poorer immunogenic capability than protein vaccines [9]. The *in silico* study evaluated the most potent epitopes from the HPV16 E5 oncoprotein, which were capable of stimulating T and B cell activities and were also entirely immunogenic through MHC class I and II [125]. This recent work identified the epitopes that are most likely to be effective as a vaccine, but in vitro and in vivo studies need to be carried out to substantiate these findings.

The use of adjuvants and its features can also have influence on the success of the vaccine. In a work using HPV16 E5 peptide vaccine in mice, activated T CD8^+^ cells and synthesis of IFN-γ were observed when administered with CpG oligodeoxynucleotides as an adjuvant [126]. CpG is a common adjuvant used to induce dendritic cell responses through TLR9 activation and the cell-mediated response in a final stage, improving the vaccine efficacy. The strong immune response caused by CpG/E5-peptide vaccine was correlated with the reduction of tumor growth [8].

Long peptides present positive features of both peptide and protein vaccines: safety, easy production and good immunogenicity. Moreover, long peptides require professional APCs, which reduce immune tolerance induction, and provide more epitopes for MHC presentation, thus increasing the response levels. HPV16 E5 long peptide together with an E6 and E7 construct was able to reduce tumor burden in mice as well as to induce a strong and prolonged immune response, with the induction of specific T CD4^+^ and CD8^+^ cells. Along with these results, the E5 + E6 + E7 vaccine had a synergistic effect and was more effective than the E5 or E6 + E7 vaccines alone. Recombinant adeno-associated virus was used as delivery system to ensure a strong immunogenicity [9].

A possible good alternative to activate key cells or to create a favorable tumor milieu to achieve an effective immune response is the cytokine modulation. Several studies have given proof of the benefits of cytokine administration or their expression modulation for induction of the host’s immune system by itself (mainly through the activation of cytotoxic cells) or by supporting other immunotherapeutic approaches [121, 122, 127].

Among the vaccine methodologies already mentioned, DNA vaccines are a promising therapy for tumors and viral infections. Key features of this methodology are: easy production with high purity and ability to induce stable expression of antigens [128]. However, this type of vaccine has a weak intrinsic potency when compared with peptide and protein vaccines [9]. Thus, the use of synergistic approaches is preferable. In a work published recently, the host immunologic activation by E5 DNA vaccine was observed in mice using the whole HPV16 E5 ORF gene and the HPV16 E5multi, a harmless version of the oncoprotein designed to express multi epitope sequences. The HPV16 E5 vaccine caused a great reduction of the tumor volume, similar to that induced by HPV16 E6 and HPV16 E7 DNA vaccines, although E6 and E7 vaccines were more effective in delaying tumor growth. E5 vaccine, unlike E6 and E7, was also able to induce T CD8^+^ cell activity causing tumor shrinkage in absence of any adjuvant [10]. In another study, the same HPV16 E5 and E5multi sequences were fused to a capsid protein sequence of the plant virus PVX that is known to induce strong immunological responses [128]. These new E5 DNA vaccines were able to induce T CD8+ response and improved antitumor activity in new murine models of ano-genital or oropharynx tumors [129].

However, other vaccine strategies for E5 remain untested. One example of these unexplored strategies is the autologous DC vaccine. Autologous monocytes were differentiated in DC in vitro and these cells were administered back in the patient after being loaded with HPV 16/18 antigens in cultured system such as E7 oncoprotein, which led to a rise in CD8^+^ T cell activity and secretion of IFN-γ. DC is known as the most potent cell for CTL induction, and the use of HPV16/18 oncoproteins as antigens for development of DC vaccines showed good results [130–132], but no trials were carried out for the E5 oncoprotein. In some cases, an additional synergistic approach can be used to improve vaccine efficacy, such as the blockade of PDL-1 [132]. A DNA vaccine comprising a gene fusion of HPV16 E6/E7 with CTLA-4 induced a greater CTL response in a mouse tumor model.

Another important example of untested strategy is the use of recombinant vaccines with bacteria as vectors applied for E5. This strategy was tested with the E7 oncogene, using *Listeria monocytogenes* (Lm) and proved to be safe in a phase-1 safety protocol. In this vaccine, E7 was fused with the non-hemolytic protein fragment listeriolysin O (LLO). Lm itself was found to induce strong CD8^+^ and CD4^+^ responses in patients with cervical cancer, and the recombinant E7/LLO antigen showed a significant degree of specific immunogenicity as well [133]. However, systemic listeriosis following vaccination was reported in a patient. Following this serious adverse event, the trial was immediately put on hold and thereafter, following consultation with the vaccine manufacturer (Advaxis), the trial was terminated early [134].

Together, these works indicate the benefit of using different immunotherapeutic approaches at the same time to act synergistically, potentializing therapeutic effects. The use of E5 by means of these strategies should provide more knowledge about E5 activity as an antigen and help in the search of an appropriate HPV-related cancer therapy. In general, E5 vaccines may represent a good choice for immunotherapeutic strategies. Although more studies are needed, E5 seems to be a valid tool to achieve a more effective therapeutic HPV vaccine, possibly integrated by check point inhibitor treatments or miRNA regulation [135]. Finally, other E5-related activities could be used in HPV-related therapy and some of them are summarized in Table 2.

**Table 2:** E5-related activities to be addressed for novel possible immunotherapy

E5 plays a key role in DNA replication and cell proliferation [21]: important for therapeutic approaches to prevent cell transformation during the initial phases of carcinogenesis or pre-neoplastic lesions.	
E5 induces the surface expression of key mitogen receptors such as EGF-R [138, 139]: important to prevent unscheduled cell proliferation and, in turn, possible generation of additional mutations.	
E5 activates COX-2 and NF-kB signaling [49]: important to avoid exacerbated inflammatory responses and expression of undesired genes, such as *c-jun* and *c-fos*.	
E5 plays a central role in promoting immunosuppression: important for interrupting antigen presentation, prevention of NK cell activity and inhibition of interferon signaling [5].	
E5 binds to EVER proteins: hampering their activities, induces a deficient immune antiviral response [19].	

## Conclusion

Both innate immunity and adaptive immunity play a crucial role in HPV-related antitumor immune response, and the aim of this review was to evaluate the state-of-the-art E5 interaction with the host’s immune system. HPV is able to modulate immune responses in infected areas through many ways, and E5 is involved in immune surveillance and evasion strategies, leading to persistent infection. Thus, this oncoprotein seems to play a central role in modulation of host’s immune system with a direct correlation with the initial stages of cervical carcinogenesis. The E5 targeted immunotherapy showed a relative success, either as peptide vaccines with adjuvants, or DNA vaccines. Several other approaches could also be explored such as the use of autologous DC administration, or bacterial vector preparations; in addition, clinical studies regarding E5-related immunotherapeutic strategies are also required. The information summarized in this review highlights the importance of this viral protein and shows that more studies must be undertaken to obtain a full understanding of the complete range of E5 activities.

## Abbreviations

Akt: Protein kinase B
AP-1: Activator protein 1
APC: Antigen presentation cell
Bap31: B-cell-associated protein 31
Bcl-2: B-cell lymphoma 2
BPV: Bovine papillomavirus
c-CBL: E3 ubiquitin-protein ligase CBL
CD1d: Cluster of differentiation 1 d
CDK2: Cyclin-dependent kinase 2
CIN: Cervical intraepithelial neoplasia
co-SMADs: Co-operating SMAD
COX: Cyclooxygenase
CREB: Cyclic adenosine monophosphate response element binding protein
CTL: Cytotoxic T lymphocytes
DC: Dendritic cell
dsRNA: Double-stranded RNA
EGF: Epithelial growth factor
EGF-R: Endothelial growth factor receptor
ELR: Region between the early and late genes
EP4: Prostaglandin E2 receptor 4
ER: Endoplasmic reticulum
ERK: Extracellular signal-regulated protein kinase
GA: Golgi apparatus
GAS: IFN-γ activated sequence
GM-1: Ganglioside-1
GM-CSF: Granulocytic-macrophage colony-stimulating factor
HLA: Human leukocyte antigen
HPV: Human Papillomavirus
hrHPV: High risk HPV
HSPG: Heparan sulphate proteoglycan
IFN-γ: Interferon γ
IKK: IκB kinase
IL: Interleukin
iNKT: Invariant natural killer T cells
IRF-1: IFN regulatory factor-1
IRFs: IFN regulatory factors
ISGF-3γ: IFN-stimulatory gene factor - 3γ
ISGs: IFN-stimulated genes
ISRE: IFN-stimulated response element
JAK: Janus kinase
KC: Keratinocytes cell
LLO: Listeriolysin O
Lm: *Listeria monocytogenes*
lrHPV: Low risk HPV
MAPK: Mitogen-activated protein kinase
MAVS: Mitochondrial antiviral signaling
MHC: Major histocompatibility complex
MICA: MHC class I polypeptide-related sequence A
MICB: MHC class I polypeptide-related sequence B
NF-1: Nuclear factor 1
NF-κB: kappa B nuclear factor
NK: Natural killer
ORF: Open Reading Frame
p300/CBP: CREB-binding protein
PGE_2_: Prostaglandin E2
PI3K: Phosphatidylinositol 3-kinase
PKR: Protein kinase RNA activated
pRB: Retinoblastoma family proteins
PRR: Pattern recognition receptors
R-SMADs: Receptor-associated SMAD
sMICA: Soluble MICA
TCR: T cell receptor
TGF-β: Transforming growth factor β
TGF-βR: Transforming growth factor β receptor
TGIF: Homeodomain protein TG-interacting factor
Th: Helper T Lymphocytes
TNFL: Tumor necrosis factor ligand
TNF-α: Tumor necrosis factor α
Treg: T-regulatory cell
Tyk: Tyrosine kinase
ULBPs: UL16-binding proteins
VEGF: Vascular endothelial growth factor
VLP: Virus-like particles
